# Thermal Scanning of Dental Pulp Chamber by Thermocouple System and Infrared Camera during Photo Curing of Resin Composites

**DOI:** 10.22037/iej.v13i2.18756

**Published:** 2018

**Authors:** Faeze Hamze, Seyed Abdolreza Ganjalikhan Nasab, Ali Eskandarizadeh, Arash Shahravan, Fatemeh Akhavan Fard, Neda Sinaee

**Affiliations:** a * Oral and Dental Disease Research Center, Kerman University of Medical Science, Kerman, Iran; *; b * Mechanical Engineering Department, Engineering School, Shahid Bahonar University, Kerman, Iran; *; c * Endodontology Research Center, Kerman University of Medical Science, Kerman, Iran *

**Keywords:** Exothermic Reaction, Infrared Camera, Light Curing Unit, Pulp Temperature, Resin Composite, Thermocouple

## Abstract

**Introduction:**

Due to thermal hazard during composite restorations, this study was designed to scan the pulp temperature by thermocouple and infrared camera during photo polymerizing different composites.

**Methods and Materials:**

A mesio-occlso-distal (MOD) cavity was prepared in an extracted tooth and the K-type thermocouple was fixed in its pulp chamber. Subsequently, 1 mm increment of each composites were inserted (four composite types were incorporated) and photo polymerized employing either LED or QTH systems for 60 sec while the temperature was recorded with 10 sec intervals. Ultimately, the same tooth was hemisected bucco-lingually and the amalgam was removed. The same composite curing procedure was repeated while the thermogram was recorded using an infrared camera. Thereafter, the data was analyzed by repeated measured ANOVA followed by Tukey’s HSD Post Hoc test for multiple comparisons (*α*=0.05).

**Results:**

The pulp temperature was significantly increased (repeated measures) during photo polymerization (*P*=0.000) while there was no significant difference among the results recorded by thermocouple comparing to infrared camera (*P*>0.05). Moreover, different composite materials and LCUs lead to similar outcomes (*P*>0.05).

**Conclusion:**

Although various composites have significant different chemical compositions, they lead to similar pulp thermal changes. Moreover, both the infrared camera and the thermocouple would record parallel results of dental pulp temperature.

## Introduction

The main backbone structure of almost all dental resin composite monomers includes carbon double bonds (C=C) which are converted to single bonds (C-C) through an exothermic reaction to produce the interconnected polymer. This exothermic reaction is started by a photo-initiator molecule that is triggered by visible blue light *via* a light curing unit (LCU) in dental clinics [1-3]. Moreover, every light source (including the LCU in dental clinics) could lead to temperature elevation in every irradiated surface [4-6]. Therefore, since dental pulp is a highly vascularized tissue that is susceptible to thermal injuries, its viability would be compromised when restoring the tooth by resin composites [7-9]. Accordingly, many previous researches were performed to evaluate the thermal changes during photo polymerization of dental composites by LCUs [1, 8, 10-12]. In order to evaluate the thermal changes beneath resin composites, most investigators incorporated thermocouple or thermometer, while sporadically, in few studies the infrared camera recorded the temperature [1, 8, 10, 12-16]. 

The thermocouple system could particularly measure the temperature of quit localized point locations. In addition, for precise recording, it needs direct contact with the object. Hence, this is categorized as an invasive method [1]. Nonetheless, despite these mentioned disadvantages, this system is currently used in many *in vitro* experiments because of its accuracy in point measurement [11-15].

**Figure 1:**
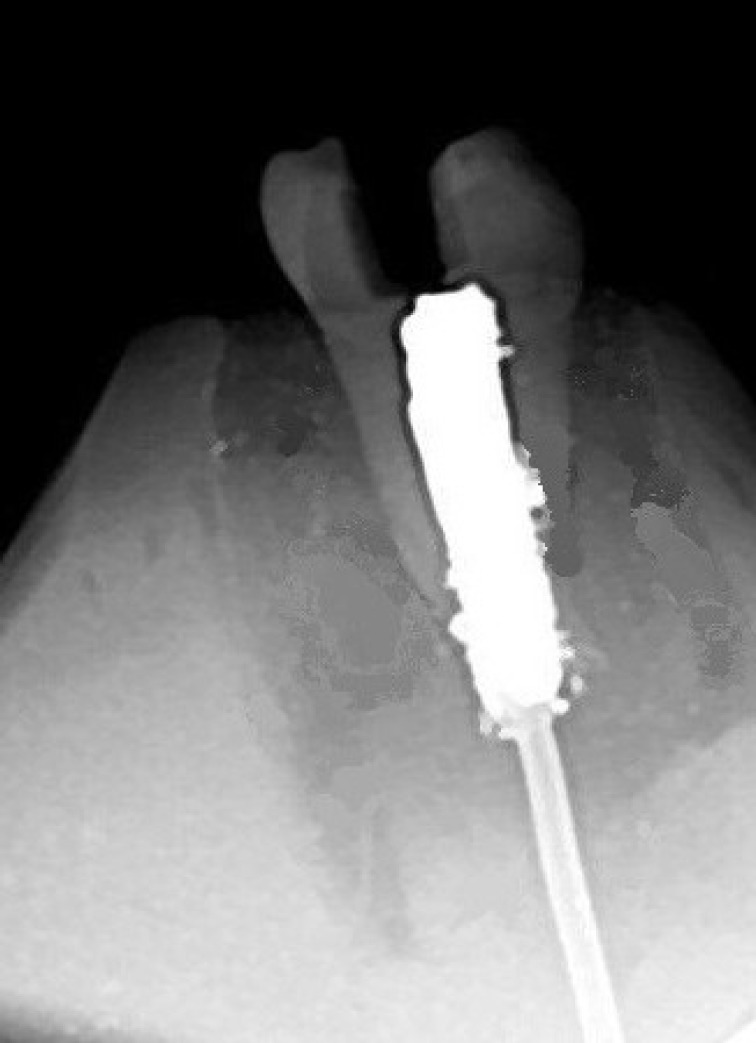
Radiographic confirmation of thermocouple in the pulp chamber

Meanwhile, the infrared thermography is a perfect reliable device that could provide two dimensional thermal images of the target surface and could have the sensitivity of 0.1^°^ C while it does not need direct contact with the object [1]. Thus, it has been frequently utilized as a modality for *in vivo* thermal analysis [8, 10].

Although the thermocouple could not be incorporated to record the temperature of the whole pulp chamber, very few studies has used their own experimental set-up system to measure the temperature of near pulp dentine junction using thermocouple [10, 17]. But supplementary investigations were always suggested.

Regarding to the ascending demand for resin composite restorations and in respect to their thermal hazard to the pulp tissue [11], justifying and comparing different modalities for recording thermal changes of the pulp chamber would be beneficial. Based on this subject, the available data regarding comparison of thermocouple and infrared camera for recording tooth temperature during composite photo curing are quite sparse. In one study, in was claimed that the thermocouple would underestimate the results comparing to infrared camera [10]; but additional studies were strongly suggested. 

Therefore, this study was designed to compare the efficacy of thermocouple and infrared camera for recording the pulp temperature when photo polymerizing different resin composites by means of different LCUs.

## Materials and Methods

***Materials and devices***

In order to assess the effect of different types and shades of resin composites, a nano-hybrid type (Tetric N-Ceram, Bulk Fill, Ivoclar Vivadent, Liechtenstein) and a micro-hybrid type (Vit-l-esence) were incorporate in this study while two shades of each commercial brand were selected. Actually, four resin composites were used Including Tetric N-Ceram shade A1 (Lot: R09966, Ivoclar-Vivadent, Schaan/Liechtenstein), Tetric N-Ceram shade A3.5 (Lot: U13037, Ivoclar-Vivadent, Schaan/Liechtenstein), Vit-l-esecnce shade PN (Lot: 1-800-552-5512, Ultradent, USA) and Vit-l-escence shade A3.5 (Lot: 1-800-552-5512, Ultradent, USA).

**Figure 2:**
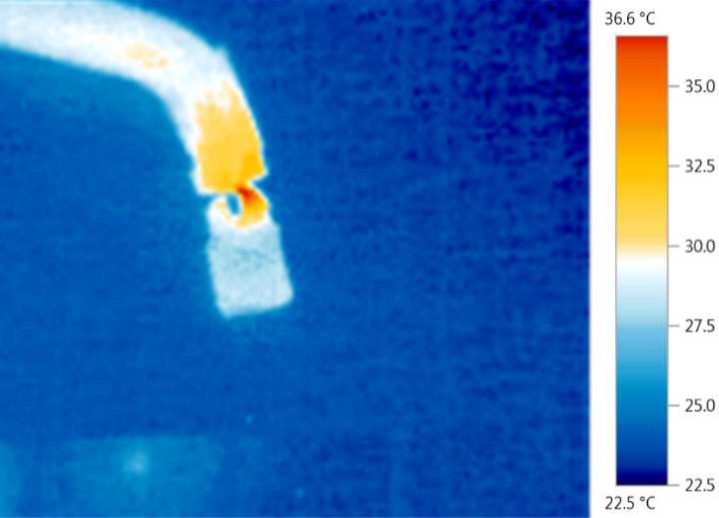
The thermal image of tooth during photo polymerization of resin composite

For comparing the efficacy of different curing devices, one type of LED (Dentamerica, LITEX, 695c,Taiwan) as well as one type of Quartz Tungsten Halogen (QTH) (Demetron LC, Kerr, Orange, CA, USA) LCU were used in the current experimentation.

Moreover, a 1 mm tip diameter K-type thermocouple (TES-1310 digital thermometer, TES electrical electronic corp., Taipei, Taiwan) along with an infrared camera (Testo 885-1, Germany) recorded the thermal changes of the samples.

***Tooth preparation***

An intact, caries free human mandibular molar were incorporated in this experiment which was stored in saline solution. At the start of the procedure, its roots were cut 3 mm below the CEJ using a diamond bur and a high speed handpiece prior to cleaning the coronal pulp chamber. Afterward, a mesial-occlusal-distal (MOD) cavity was prepared on the crown adjusting bucco lingual dimension as 2 mm while its depth was set as the remaining dentine thickness between the mesial pulp horn and the pulpal wall was 0.5 mm (assessed by a gage crown).

***Incorporating thermocouple***

The thermocouple tip was inserted into the pulp chamber of the prepared tooth, touching the roof of the chamber, and its surrounding was filled by condensing some amount of amalgam (ANA 2000, Nordiska Dental, Sweden) to fill the pulp cavity. Noticeably, the amalgam played two important roles: stabilization of the thermocouple tip and it was served as a heat transferring medium to uniformly distribute the generated heat in pulp chamber since amalgam is categorized as a very good heat conductor [17]. Ultimately, the remaining portion of the root besides the extension of the thermocouple tip was mounted in a silicon block to fix the setup model (Figure 1). 

**Figure 3:**
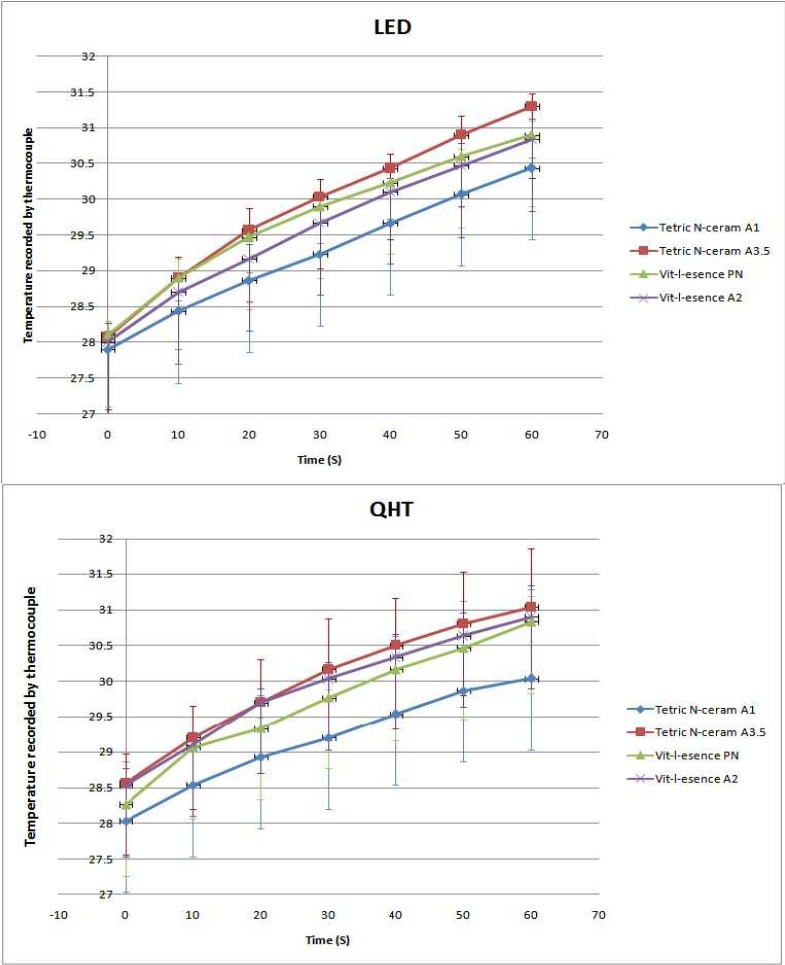
The mean recorded temperature ± SD using thermocouple when curing different composites by either LED or QTH light curing units

Thereafter, 1 mm thick increment of either composite resins were condensed into the prepared cavity without application of neither acid etching nor resin bonding (the composite could be easily removed from the cavity after curing and the same tooth was repeatedly fill again) [18]. Subsequently, the inserted composite layer was light cured for 60 sec by either type of LCUs while the LCU tip was directly attached to the occlusal surface of the tooth. Simultaneously, at every 10 sec intervals the displayed temperature was recorded. This procedure was triplicated (*n*=3) in either of eight subgroups (incorporating four types of composites and two types of LCUs lead to eight experimental subgroups). 

***Incorporating infrared camera***

After completion of the thermal measurements with thermocouples, the same tooth was sectioned bucco-lingually using a low-speed diamond saw (Isomet, Buehler, Ltd., Lake Bluff, IL, USA) and the mesial section were selected for the infrared thermography analysis (since the mesial pulp horn is the closest part of the pulp to the pulpal floor of the prepared cavity). The previously inserted amalgam was removed from the pulp cavity using a high speed headpiece by an expert clinician under 2.5× magnification loop.

**Figure 4:**
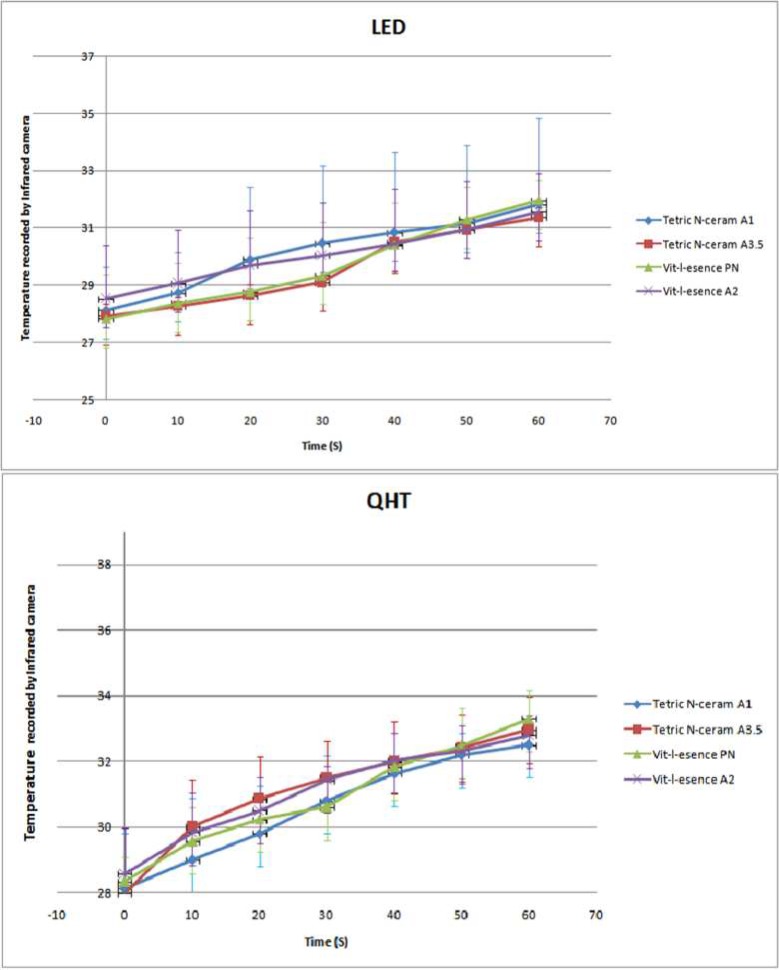
The mean recorded temperature ± SD using infrared camera when curing different composites by either LED or QTH light curing units

Thereafter the exactly same protocol was repeated for inserting and curing the resin composites. However, since the tooth was hemisected, half of each LCUs tips were covered by an aluminum foil. Similarly, at every 10 sec intervals, an image was captured by the infrared camera which was fixed in a 30 cm distance. Subsequently, the thermal images were imported to an image processing and analyzing software (Adobe Photoshop, CS5, Adobe Systems Inc., San Jose, CA, USA) in order to record the highest temperature at the pulp-dentine junction (Figure 2). 

Data were analyzed using repeated measures ANOVA followed by Tukey’s HSD post hoc test for multiple comparisons (*α*=0.05).

## Results

The mean pulp temperature recorded by thermocouple and infrared camera are displayed in Figures 3 and 4 respectively. As can be seen in these diagrams, the temperature has been elevated in all subgroups and by statistical analysis it was revealed that the pulp temperature significantly increased from 0 up to 60 sec after light curing of the composites (*P*=0.000). Moreover, there were no interactions between neither of the LCUs, thermal recorders (thermocouple or infrared camera) and the composite type (*P*=0.36). 

Meanwhile, the *P*-values related to the pairwise comparison of different subgroups are demonstrated in Table 1. Accordingly, three main results are obtained from these comparisons: First of all, there is no significant difference between composites cured by the same LCU and scanned by the same device. Secondly, two types of LCUs showed the same results. Finally, scanning of the tooth temperature by both the thermocouple and the infrared devices lead to the same heat trend.

## Discussion

The results of the current study revealed that there was no significant difference between the recorded results of thermocouple and infrared camera during photo polymerization of resin composites. However, the pulp temperature exacerbated only around 3-4^°^ C in both groups that could be considered safe clinically. 

This finding is in contrast with Bouillaguet *et al.* [10], who stated that the thermocouples underestimated the heat comparing to infrared camera. Moreover, they found that the intra pulp temperature was raised around 2-5 ^°^C that is quite similar to our range. However, for intimate contact of thermocouple tip with dentine they used sodium chloride solution because it conducts the heat energy as like as dentine tissue [19]; while we filled the pulp chamber by a conductor material (amalgam) to record the mean temperature diffused in the whole pulp chamber. They also claimed that the highest temperature value was observed on the external surface of the examined tooth [10], our infrared maps also showed the peak temperature point on the cuspal incline adjacent to the LCU (according to the colored map) although this point was not included in the aim of this study and we did not measure the exact temperature.

Interestingly, our result does not present a bad scenario for the temperature rise during photo polymerization because we observed only 3-4^°^C increase in pulp temperature. However, according to published literatures, in healthy pulp as the temperature arise at least 5.2^°^C, the pulp necrosis started in 15% of animal small teeth [20]. 

Nevertheless, some previous researches indicated higher heat emission. In view of that, Al-Qudah *et al.* [1] reported 36.3 ^°^C rise in pulp temperature after 5 sec of curing 2-mm thick dental composite employing a QTH lamp. Moreover, in a clinical study on maxillary incisors, Hussey *et al*. [8] also claimed about 12°C elevation in tooth temperature during photo curing of resin composite while the tooth was scanned by an infrared system. 

Recently, Kim *et al.* [12] performed an *in vitro* study in which the ascending temperature diagrams were represented during polymerization of resin composite using thermocouples in an extracted tooth. Although they reported very great heat elevation in top, bottom and middle layers of composite, the temperature was increased very gradually (only about 3 to 5.5^°^C) at the pulpal side of the remaining 0.5 mm dentin within the pulp chamber [12]. Therefore, it could be assumed that the remaining dentin, even in very thin layers, imparts a very good insulator against heat transfer [1, 21]. Obviously, in most clinical situations, at least a very thin layer of dentin would exist in pulpal wall following tooth preparation and caries excavation. 

**Table 1:** Tukey HSD P-values related to pairwise comparison of all subgroups

	**T,A1,Q**	**T,A3.5,L**	**T,A3.5,Q**	**T,PN,L**	**T,PN,Q**	**T,A2,L**	**T,A2,Q**	**I,A1,L**	**I,A1,Q**	**I,A3.5,L**	**I,A3.5,Q**	**I,PN,L**	**I,PN,Q**	**I,A2,L**	**I,A2,Q**
**T,A1,L**	1.0	1.0	1.0	1.0	1.0	1.0	1.0	0.98	0.93	1.0	0.60	1.0	0.75	1.0	0.62
**T.A1.Q**		1.0	0.99	1.0	1.0	1.0	1.0	0.99	0.91	1.0	0.55	1.0	0.70	0.99	0.57
**T,A3.5,L**			1.0	1.0	1.0	1.0	1.0	1.0	1.0	1.0	0.97	1.0	0.99	1.0	0.97
**T,A3.5,Q**				1.0	1.0	1.0	1.0	1.0	1.0	1.0	0.98	1.0	0.99	1.0	0.99
**T,PN,L**					1.0	1.0	1.0	1.0	0.99	1.0	0.93	1.0	0.97	1.0	0.94
**T,PN,Q**						1.0	1.0	1.0	0.99	1.0	0.91	1.0	0.97	1.0	0.93
**T,A2,L**							1.0	1.0	0.99	1.0	0.85	1.0	0.94	1.0	0.86
**T,A2,Q**								1.0	1.0	1.0	0.97	1.0	0.99	1.0	0.97
**I,A1,L**									1.0	1.0	0.99	1.0	1.0	1.0	0.99
**I,A1,Q**										0.99	1.0	0.99	1.0	1.0	1.0
**I,A3.5,L**											0.83	1.0	0.93	1.0	0.85
**I,A3.5,Q**												0.92	1.0	0.99	1.0
**I,PN,L**													0.97	1.0	0.93
**I,PN,Q**														0.99	1.0
**I,A2,L**															0.99

T: thermocouple, I: infrared, A1: Tetric N-Ceram composite shade A1, A3.5: Tetric N-Ceram composite shade A3.5, PN: Vit-l-esence composite shade PN, A2: Vit-l-esence composite shade A2, Q: QTH light cure unit, L: LED light cure unit

Overwhelmingly, it can be concluded that although in many *in vitro* studies the photo curing process of resin composites led to heat emission, it seems this phenomenon does not have any considerable clinical outcome on the pulp tissue regarding the dentine barrier between dental composite and the pulp.

## Conclusion

Under the limitation of this study, it was revealed that there is no significant difference among thermocouple device and infrared camera for scanning the pulp temperature during photo polymerization of dental resin composites. Moreover, when there is 0.5 mm dentine barrier, the pulp temperature raised only 3-4^° ^C that is not considered as a worse-case clinical scenario.
